# Do Communication Patterns Affect the Association between Cognitive Impairment and Hearing Loss among Older Adults in Vietnam?

**DOI:** 10.3390/ijerph18041603

**Published:** 2021-02-08

**Authors:** Tran Dai Tri Han, Keiko Nakamura, Kaoruko Seino, Vo Nu Hong Duc, Thang Van Vo

**Affiliations:** 1 Department of Global Health Entrepreneurship, Division of Public Health, Graduate School of Medical and Dental Sciences, Tokyo Medical and Dental University, Tokyo 113-8519, Japan; trandaitrihan@hueuni.edu.vn (T.D.T.H.); seino.ith@tmd.ac.jp (K.S.); 2 Faculty of Public Health, Hue University of Medicine and Pharmacy, Hue University, Hue 530000, Vietnam; vnhduc@hueuni.edu.vn (V.N.H.D.); vovanthang147@hueuni.edu.vn (T.V.V.); 3 The Institute for Community Health Research, Hue University of Medicine and Pharmacy, Hue University, Hue 530000, Vietnam

**Keywords:** communication tools, cognitive impairment, older adults, hearing loss, social interaction

## Abstract

This study examined the prevalence of cognitive impairment among older adults in central Vietnam and the roles of communication (with or without communication devices) in the association between cognitive impairment and hearing loss. This cross-sectional study was performed on 725 randomly selected community-dwelling older adults aged ≥60 years from Thua Thien Hue province, Vietnam. Participants attended a face-to-face survey. Sociodemographic characteristics, social interaction with or without communication devices, health status and cognitive function using the Mini-Mental State Examination were reported. Ordinal logistic regression analysis was performed to quantify the association between hearing loss and cognitive function by frequency of communication with and without devices. Mild and severe cognitive impairment had prevalence rates of 23.6% and 19.3%, respectively. Cognitive impairment was more prevalent among older adults with hearing-loss, vision loss and difficulties with instrumental activities of daily living (IADL). The association between hearing loss and cognitive impairment was not significant when older adults had frequent communication with others using devices. This study presented the relatively high prevalence of cognitive impairment in community-dwelling older adults in Vietnam. Frequent communication using devices attenuated the association between hearing loss and cognitive impairment.

## 1. Introduction

Promotion of cognitive health has been a public health priority in rapidly aging societies considering its impact not only on older adults’ quality of life, but also on their families, health care system and economy [1]. Vietnam is one of the countries with fastest rate of aging in the world [2]. The number of people aged ≥65 years in Vietnam was 7.4 million in 2019, accounting for 7.7% of population [3], which is expected to increase to 18.1% by 2049 [4].

Hearing loss, the most prevalent sensory deficit affecting about one in three adults aged over 65 years [5], has not been a priority in cognitive impairment risk management for long [6]. However, cohort studies have recently showed that even mild levels of hearing loss increase the longer-term risk of cognitive decline [7,8]. However, reported results on the association between cognitive impairment and hearing loss are conflicting [9,10] and the underlying mechanism to clearly explain these two associations is not yet established.

Hearing loss leads to difficulties in communication and creates barriers in interaction [11,12]. Social isolation, defined as having a small social network or a lack of close relationships or sources of social support [13], is one of the factors that relate both with cognitive impairment and hearing loss [14,15]. The development of information technology has diverted ways of communication from simple telephone calling to video calls or text messaging application, which allows declined visual and hearing function to be mutually complemented [16]. Easy-to-use options, including modulating pitch and amplitude on communication devices, enable older adults to compensate peripheral functional deficit of age related hearing loss [17]. In addition, communication device usage makes up for face-to-face communication limitation due to changes in family structure and lifestyles [18]. Considering the interrelationships of cognitive impairment, hearing loss, and interpersonal communication, the question emerges how usage of communication tools may affect the association between cognitive impairment and hearing loss.

In Vietnam, with the rapid development of information and communication technology, telephone and internet access have become more accessible and affordable. The percentage of internet users per 100 inhabitants in Vietnam exceeds 50%, and the number of mobile cellular telephone subscribers is equal to the size of the Vietnamese population [3,19]. In 2019, 91.7% of households owned phone or mobile phone or tablet (comparing to 45.7% in 2009) [3,20]. In addition, communication applying these technologies into usage of smartphones or social media has been penetrated even among older adults in communities where face to face communication with their children, relatives, and close friends is limited due to changes in family structure and lifestyles [21].

To date, there were few primary studies in Vietnam focused on the cognitive impairment of older adults. These studies mainly investigated epidemiological aspects of cognitive impairment. The previous Vietnam reports showed the prevalence of cognitive impairment ranged from 29% to 48% [22,23,24]. However, a lack of study included mild cognitive impairment (MCI), an early stage of dementia. Moreover, though much research has documented that hearing loss was associated with cognitive impairment in high-income countries, a minimal number of studies have been done in low- and middle-income countries, including Vietnam, and few have focused on the role of communication patterns. A better understanding of communication patterns’ role in the association between hearing loss and cognitive impairment would provide valuable insights into potential approaches in preventing or delaying cognitive decline progress.

This study was performed to estimate the prevalence of cognitive impairment among older adults in central Vietnam and explore the effects of the use of communication devices on the association between hearing loss and cognitive impairment.

## 2. Methods

### 2.1. Participants

This cross-sectional investigation was conducted in Thua Thien Hue province in the central region of Vietnam between June and July 2018. A total of 725 community-dwelling adults aged ≥ 60 years were selected by two-stage random sampling. In stage one of the sampling process, the population was divided into rural and urban areas. Two of 27 quarters in Hue city (urban areas) and three of 19 quarters of Phu Vang district (rural areas) were randomly selected. In stage two, the participants were proportionate randomly selected from lists of all potential participants in the five quarters. Participants who were unable to communicate were excluded.

Because the standardized cutoffs for cognitive impairment were developed primarily in Western countries and no normative studies have been conducted in Vietnam, use of the Western cutoffs is potentially problematic due to the inclusion of illiterate participants who would not be able to respond to questions requiring reading and writing ability. Therefore, participants who were illiterate were excluded (*n* = 81) to reduce misclassification errors in the present study. Evaluation of cognitive function by using the Mini Mental State Examination (MMSE) determined 81 potential participants ineligible to continue with the study. The remaining 644 literate participants were interviewed and their information analyzed.

### 2.2. Measurements

The study participants attended a face-to-face interview with interviewers who were public health specialists with psychiatric knowledge. Questions designed in English were converted to Vietnamese by a forward and back translation process.

#### 2.2.1. Sociodemographic and Types of Interaction

In addition to age and sex, level of education was categorized into elementary school or lower, secondary or high school, and university or higher. Living area was categorized as rural or urban. Living with spouse, and children were categorized as “yes” or “no.” Financial strain was categorized as “no” when participants had enough money for their daily needs over the past month, or “yes” if they lacked money for daily needs.

Interaction using communication devices was defined as the use of tools of telephone, e-mail, or social media to communicate with family and others. Interaction not using communication devices was defined as face-to-face direct interactions with family and others at social gatherings, such as going out together or visiting each other’s homes. Both interactions using and not using communication devices were classified as frequent (≥2 times per week) or infrequent (<2 times per week).

#### 2.2.2. Health Status and Lifestyles

##### Cognitive Function

Cognitive function was evaluated using the MMSE [25], a paper-based test that is a commonly used standard instrument for detection of cognitive impairment [26,27]. The MMSE includes two parts, the first of which requires vocal responses and covers orientation to time and place, word repetition and recall, and attention, while the second part tests the ability to name objects, follow verbal and written commands, write sentences, and copy complex polygons. The MMSE total score ranges from 0 to 30, with higher scores indicating better cognitive function. Cognitive function evaluated by MMSE was divided into three categories: MMSE 0–23, MMSE 24–27, and MMSE 28–30 based on cutoff points of 23/24 and 27/28 reported to distinguish different older adults’ cognition [27,28,29]. 

##### Sensory Function

A short questionnaire to assess self-perceived hearing and vision loss was administered at the interviews. In community surveys to cover a large population, this method is widely used, and several studies have shown that self-rated hearing impairment is correlated with audiometric measures in older adults [30,31]. Participants were asked to rate their hearing and vision ability as: no difficulty, difficult, or very difficult. Participants with hearing or vision ability classified as difficult or very difficult were categorized as having hearing or vision loss. 

##### Instrumental Activities of Daily Living (IADL)

Eight items of the Lawton Instrumental Activities of Daily Living (IADL), i.e., using a telephone, shopping, food preparation, housekeeping, doing laundry, using transportation, taking medications, and financial behavior, were used to assess the ability of older adults participants to perform daily tasks [32]. Difficulty with IADL was defined as the inability to perform at least one of the above items independently [33].

##### Presence of Chronic Disease

The presence of chronic disease (angina, asthma, arthritis, cataracts, chronic obstructive pulmonary disease, diabetes, hypertension) was categorized as no, one, and more than one chronic disease based on self-reporting of clinical diagnosis history. 

##### Smoking

Current smoking status was categorized as yes or no.

### 2.3. Statistical Analysis

The frequencies of demographic and socioeconomic characteristics, interaction types, cognitive function, health status, living arrangement, and lifestyles of participants were calculated. The chi-square test was used to compare the levels of cognitive impairment according to the frequencies of interaction types, sociodemographic characteristics, health status, and lifestyles of participants. Ordinal logistic regression analysis was performed to quantify the associations between hearing loss and cognitive function outcomes according to the frequencies of interaction types, with adjustments for sociodemographic characteristics, health status, living arrangement, and lifestyle.

Ordinal logistic regression is the best fit when the dependent variable is ordinal and can be ordered in a natural way [34]. Odds ratio (OR) >1 indicates exposure associated with higher odds of decreased cognitive function. 

Statistical analyses were performed using IBM SPSS Statistics version 25.0 (IBM, Armonk, NY, USA). In all analyses, *p* < 0.05 was taken to indicate statistical significance.

## 3. Results

The characteristics of participants are shown in Table 1. The total number of study participants included in the statistical analysis was 644 literate adults aged 60 years and above, nearly half of whom were aged 60–69 years old, the majority were women (54.5%), and more than a half had elementary school or lower educational level. Most subjects lived with a spouse or/and children. Self-rated hearing loss was documented in 21.9% of participants. There was no significant difference in the characteristics of participants between the original sample size (*n* = 725) and the sample of only literate participants (*n* = 644).

Table 2 presents the levels of cognitive function according to the type of interactions, and the characteristics of the participants. Overall, 124 (19.3%) participants were categorized as having severe cognitive impairment (MMSE 0–23), 152 (23.6%) had mild cognitive impairment (MMSE 24–27), and 368 (57.1%) had normal cognitive function (MMSE 28–30). The prevalence rates of MMSE group scores were significantly different between participants with different frequencies of interaction types and hearing function (*p* < 0.05).

Table 3 shows the results of multivariable ordinal regression analysis of the association between hearing loss and cognitive function, stratified according to the frequencies of interaction using and not using communication devices. In the all combined model (model 1), cognitive impairment was associated with hearing loss, vision loss, being female, age over 80, lower education, current smoking, and difficulty with IADL. Model 2 showed association of hearing loss with cognitive impairment in participants with infrequent interactions using devices (OR: 2.23, 95% CI: 1.26–3.93 *p* = 0.006). However, association between hearing loss and cognitive impairment was attenuated in model 3, and this relationship was not significant in participants with frequent interactions using communication devices (OR: 1.58, 95% CI: 0.85–2.96, *p* = 0.148). In models 4 and 5, self-rated hearing loss showed a significant association with cognitive impairment in participants with both infrequent and frequent interactions not using communication devices. Furthermore, vision loss, current smoker, and IADL difficulty showed significant associations with cognitive impairment regardless the frequencies of the interaction types.

## 4. Discussion

Severe (MMSE 0–23) and mild (MMSE 24–27) cognitive impairment (MCI) had prevalence rates of 19.3% and 23.6%, respectively, in our study population. The results presented here also suggested that the association hearing loss and cognitive function varied according to the frequencies and ways of communication. In particular, hearing loss was associated with decreased cognitive function among participants with infrequent interactions using devices and among those who had face-to-face interaction without devices regardless of the frequency. In contrast, frequent interactions using communication devices attenuated the association between hearing loss and cognitive impairment. 

### 4.1. Prevalence of Mild and Severe Cognitive Impairment

In the present study, the combined prevalence of mild and severe cognitive impairment was 42.9%, highlighting the public health impacts of these conditions and the need for national strategies to prevent cognitive impairment in older adults [35].

Based on 10 studies in eight countries the Cohort Studies of Memory in an International Consortium (COSMIC) showed that the prevalence of MCI ranged from 2.1% to 20.7% [36]. The COSMIC used the same criterion for MCI as in the present study (MMSE 24–27), but participants were people aged ≥ 65 years and participants with dementia were excluded. 

With regard to the prevalence of severe cognitive impairment, MMSE score 0–23 was used as the criterion to detect dementia and had sensitivity of 0.89 (95% CI, 0.85 to 0.92) and specificity of 0.89 (95% CI, 0.85 to 0.93) [26]. Using the same criterion of MMSE 0–23, but with some differences in methodologies and study populations, other studies in Vietnam reported severe cognitive impairment prevalence rates of 29–48% [22,24].

Furthermore, in our study, the combined prevalence of mild and severe cognitive impairment at 42.9% highlighted the public health impact of these conditions and the urgency for conducting national strategies to prevent or delay cognitive impairment in older adults [35].

### 4.2. Diverse Association of Hearing Loss and Cognitive Function According to Interaction’s Types

The results of the present study showed cognitive impairment was more prevalent among older adults with hearing loss. Hearing loss has been recently recognized as a risk factor for dementia [6,37,38]. Hearing loss may be causally associated with cognitive impairment via increased cognitive load, changes in brain structure and function, and increased social isolation [14].

Our results showed that frequent interactions with family and others at social gatherings using communication devices attenuated the association between hearing loss and cognitive impairment. This may be explained by the attenuating effects of frequent interactions with communication supportive devices on social isolation in older adults with hearing loss, while the infrequent interactions or both frequent and infrequent interactions without using communication devices showed no such effect.

Direct interactions with family and others at social gatherings, such as going out together or visiting each other’s homes not applying any communication device, are traditional ways to provide or receive social support. However, the direct interactions could bring challenges for older adults sometimes. Frequent interactions and dense social networks sometimes result in intrusive support, overwhelming advice and interference, and may exacerbate stress [39,40]. In particular, older adults may perceive well-intentioned support efforts from family or friends as control. Although such well-intentioned support can have beneficial effects on health outcomes, it can potentially lead to the development of interpersonal conflict and stress [39,41]. These aspects of the direct interactions without communication technology support may become more severe in older adults with hearing loss with partly limited communication ability.

In contrast, interactions involving the use of communication devices, such as telephone, e-mail, or social media, can somewhat offset the limitations of the direct interactions. Communication using devices are much easier to connect to a selected person regardless of time and geographical barriers, thus allowing older adults to gain access to the support that they need. In addition, with high rates of urbanization, older adults may have limited choice of living with a “significant family member or others.” Indirect interactions with use of indirect means of communication allow older adults to choose the people with whom they connect. Although these selected individuals cannot provide as much instrumental support as the people who live with older adults, but they have the advantage of potentially providing better emotional support [42]. In addition, having the ability to use indirect means of communication, such as telephone, is beneficial for the self-efficacy of older adults [43]. 

In Vietnam, with the rapid development of information and communication technology, telephone and internet access have become more accessible and affordable. The percentage of internet users per 100 inhabitants in Vietnam exceeds 50%, and the number of mobile cellular telephone subscribers is equal to the size of the Vietnamese population [3,19]. In the present study, 83% of older adults had the ability to use the telephone at different levels. These observations highlight the significant advantages of public health intervention programs based on interaction using communication devices. Noteworthy, communication means should be particularly designed for older adults who may have cognitive and functional limitations. Keeping in mind that even though some older adults do not have their own communication devices or face difficulties in learning how to use them, their housemates can operate communication devices for them.

The major strengths of this study included the use of population-based representative data, face-to-face interview, and standard data collection tools. Ordinal logistic regression allowed us to examine three levels of cognitive function as a dependent variable, including MCI, which is an early stage of cognitive decline that may have potential benefits for early preventive interventions. The results of this study revealed the critical role of interaction using communication devices, including telephone, mail, and online social networks, in modifying the association between hearing impairment and cognitive function. 

This study had several limitations. First, due to its cross-sectional design, the results of associations of variables could not determine causal relationships. Further longitudinal studies are required to make causal inferences. Second, our results may have been subject to recall bias. If necessary, demographic information was double-checked with family members of the older adults included in the study. Third, the study relied on a self-reported measure of hearing loss. Our results were interpreted on the basis of understanding that self-rated hearing impairment is correlated with audiometric measures in older adults [31]. However, this correlation is still a matter of debate for cases of mild hearing impairment. Fourth, validity of MMSE in Vietnamese version to evaluate cognition of the Vietnamese population would be carefully examined in a future study. In addition, as MMSE includes two questions that evaluate reading and writing ability, illiterate participants (*n* = 81) were excluded from the analytical statistics to reduce misclassification of cognitive impairment by MMSE. This exclusion was helpful in analyzing the frequency of use of interaction using communication devices that require reading and writing ability, such as e-mail and social media. Therefore, our results should be interpreted in the context of the exclusion of illiterate participants.

Further longitudinal studies are required to elucidate the benefit of communication using devices on the association between hearing loss and cognitive impairment. Both quantitative and qualitative study designs are recommended. For example, a quantitative study could measure the impact of the specific means of communication on cognitive function in the specific context. A qualitative study could measure the advantages and disadvantages of using different means of communication from either perspective of older adults or their caregivers.

## 5. Conclusions

The association between cognitive impairment and hearing loss of older adults varied according to frequencies and ways of communication. Results of a study conducted in one of the world’s fastest aging societies, Vietnam, showed that frequent communication using devices attenuated the association between hearing loss and cognitive impairment. These results suggested that fitted communication methods for older adults whose sensory functions declined with aging is vital to maintain their cognitive function.

## Figures and Tables

**Table 1:** Characteristics of study participants included in the analysis (*n* = 644).

Variables	Number of Subjects	(%)
Demographic and socio-economic status		
Sex		
Men	293	(44.5)
Women	351	(54.5)
Age group		
60–69	313	(48.6)
70–79	186	(28.9)
≥80	145	(22.5)
Education		
Elementary school or lower	337	(52.3)
Secondary or high school	251	(39.0)
University or higher	56	(8.7)
Financial strain		
No	381	(59.2)
Yes	263	(40.8)
Living area		
Rural	372	(57.8)
Urban	272	(42.2)
Living with spouse		
No	168	(26.1)
Yes	476	(73.9)
Living with children		
No	221	(34.3)
Yes	423	(65.7)
Health status and lifestyles		
Vision loss		
No	343	(53.3)
Yes	301	(46.7)
Hearing loss		
No	503	(78.1)
Yes	141	(21.9)
IADL		
Independence in IADL	382	(59.3)
Difficulty with IADL	262	(40.7)
Presence of chronic disease		
None	189	(29.3)
One chronic disease	246	(38.2)
More than one chronic disease	209	(32.5)
Current smoking		
Yes	157	(24.4)
No	487	(75.6)
Types of interaction		
Interaction using communication devices ^1^		
Infrequent (<2 times per week)	306	(47.5)
Frequent (≥2 times per week)	338	(52.5)
Interaction not using communication devices ^2^		
Infrequent (<2 times per week)	330	(51.2)
Frequent (≥2 times per week)	314	(48.8)

^1^ Interaction using communication devices was defined as the use of tools of telephone, e-mail, or social media to communicate with family and others; ^2^ Interaction not using communication devices was defined as face-to-face direct interactions with family and others at social gatherings, such as going out together or visiting each other’s homes; IADL, Instrumental Activities of Daily Living.

**Table 2:** Cognitive function levels according to types of interactions and the characteristics of participants ^1^.

Variables	No. (%) by Cognitive Impairment			
	MMSE 0–23 (Severe) (*n* = 124)	MMSE 24–27 (Mild) (*n* = 152)	MMSE 28–30 (Normal) (*n* = 368)	*p*-Value
Types of interaction				
Interactions using communication devices ^2^				
Infrequent (<2 times per week)	80 (26.1)	71 (23.2)	155 (50.7)	<0.001
Frequent (≥2 times per week)	44 (13.0)	81 (24.0)	213 (63.0)	
Interactions not using communication devices ^3^				
Infrequent (<2 times per week)	76 (23.0)	74 (22.4)	180 (54.5)	0.045
Frequent (≥2 times per week)	48 (15.3)	78 (24.8)	188 (59.9)	
Demographic and socioeconomic status				
Sex				
Women	80 (22.8)	92 (26.2)	179 (51.0)	0.002
Men	44 (15.0)	60 (20.5)	189 (64.5)	
Age group				
60 = 69	31 (9.9)	70 (22.4)	212 (67.7)	<0.001
70 = 79	31 (16.7)	45 (24.2)	110 (59.1)	
≥80	62 (42.8)	37 (25.5)	46 (31.7)	
Education				
Elementary school or lower	99 (29.4)	96 (28.5)	142 (42.1)	<0.001
Secondary or high school	22 (8.8)	46 (18.3)	183 (72.9)	
University or higher	3 (5.4)	10 (17.9)	43 (76.8)	
Financial strain				
No	57 (15.0)	88 (23.1)	236 (61.9)	0.002
Yes	67 (25.5)	64 (24.3)	132 (50.2)	
Living area				
Rural	88 (23.7)	90 (24.2)	194 (52.2)	0.002
Urban	36 (13.2)	62 (22.8)	174 (64.0)	
Living with spouse				
No	48 (28.6)	48 (28.6)	72 (42.9)	<0.001
Yes	76 (16.0)	104 (21.8)	296 (62.2)	
Living with children				
No	40 (18.1)	55 (24.9)	126 (57.0)	0.791
Yes	84 (19.9)	97 (22.9)	242 (57.2)	
Health status and lifestyles				
Hearing loss				
No	71 (14.1)	113 (22.5)	319 (63.4)	<0.001
Yes	53 (37.6)	39 (27.7)	49 (34.8)	
Vision loss				
No	37 (10.8)	66 (19.2)	240 (70.0)	<0.001
Yes	87 (28.9)	86 (28.6)	128 (42.5)	
Current smoking				
Yes	38 (24.2)	43 (27.4)	76 (48.4)	0.036
No	86 (17.7)	109 (22.4)	292 (60.0)	
Presence of chronic disease				
No	28 (14.8)	45 (23.8)	116 (61.4)	0.045
One chronic disease	43 (17.5)	66 (26.8)	137 (55.7)	
More than one	53 (25.4)	41 (19.6)	115 (55.0)	
IADL				
Independence in IADL	28 (7.3)	74 (19.4)	280 (73.3)	<0.001
Difficulty with IADL	96 (36.6)	78 (29.8)	88 (33.6)	

^1^ Percentages may not total 100 due to rounding; ^2^ Interaction using communication devices was defined as the use of tools of telephone, e-mail, or social media to communicate with family and others; ^3^ Interaction not using communication devices was defined as face-to-face direct interactions with family and others at social gatherings, such as going out together or visiting each other’s homes; MMSE, Mini-Mental State Exam; IADL, Instrumental Activities of Daily Living.

**Table 3:** Multivariable ordinal logistic regression analyses of the associations between hearing loss and cognitive function.

Variables	Model 1 All (*n* = 644)		Interaction Using Communication Devices ^1^				Interaction Not Using Communication Devices ^2^			
			Model 2 Infrequent (<2 Times per Week) (*n* = 306)		Model 3 Frequent (≥2 Times per Week) (*n* = 338)		Model 4 Infrequent (<2 Times per Week) (*n* = 330)		Model 5 Frequent (≥2 Times per Week) (*n* = 314)	
	OR (95% CI)	*p*-Value	OR (95% CI)	*p*-Value	OR (95% CI)	*p*-Value	OR (95% CI)	*p*-Value	OR (95% CI)	*p*-Value
Health status and lifestyles										
Self-rated hearing loss										
No	1		1		1		1		1	
Yes	1.96 (1.30–2.95)	0.001	2.23 (1.26–3.93)	0.006	1.58 (0.85–2.96)	0.148	1.81 (1.02–3.21)	0.042	1.95 (1.05–3.60)	0.034
Self-rated vision loss										
No	1		1		1		1		1	
Yes	2.02 (1.41–2.88)	<0.001	1.97 (1.18–3.31)	0.010	2.07 (1.24–3.45)	0.006	2.23 (1.34–3.70)	0.002	1.74 (1.02–2.96)	0.041
IADL										
Independence in IADL	1		1		1		1		1	
Difficulty with IADL	2.76 (1.88–4.05)	<0.001	3.67 (2.08–6.48)	<0.001	2.02 (1.16–3.52)	0.013	3.32 (1.88–5.88)	<0.001	2.48 (1.44–4.26)	0.001
Presence of chronic disease										
No	1		1		1		1		1	
One chronic disease	1.27 (0.83–1.95)	0.278	1.28 (0.69–2.40)	0.431	1.40 (0.75–2.61)	0.295	1.44 (0.77–2.69)	0.252	1.01 (0.55–1.84)	0.976
More than one	1.06 (0.68–1.65)	0.812	1.10 (0.57–2.10)	0.778	1.17 (0.61–2.26)	0.630	1.65 (0.89–3.08)	0.113	0.64 (0.33–1.25)	0.191
Current smoking										
No	1		1		1		1		1	
Yes	2.60 (1.70–3.97)	<0.001	2.44 (1.32–4.51)	0.004	2.58 (1.42–4.69)	0.002	2.63 (1.39–4.99)	0.003	2.68 (1.50–4.79)	0.001
Demographic and socioeconomic status										
Sex										
Women	1		1		1		1		1	
Men	0.54 (0.36–0.82)	0.004	0.52 (0.29–0.94)	0.031	0.60 (0.33–1.08)	0.087	0.54 (0.30–0.98)	0.044	0.50 (0.28–0.91)	0.023
Age group										
60 = 69	1		1		1		1		1	
70 = 79	1.12 (0.74–1.71)	0.589	1.34 (0.73–2.47)	0.344	0.94 (0.51–1.72)	0.842	0.92 (0.50–1.67)	0.775	1.28 (0.69–2.36)	0.434
≥80	2.27 (1.73–4.43)	<0.001	3.36 (1.72–6.59)	<0.001	2.20 (1.12–4.33)	0.022	2.37 (1.18–4.76)	0.015	3.04 (1.56–5.91)	0.001
Education										
Elementary school or lower	1		1		1		1		1	
Secondary or high school	0.40 (0.27–0.60)	<0.001	0.48 (0.27–0.85)	0.012	0.30 (0.17–0.54)	<0.001	0.33 (0.19–0.58)	<0.001	0.46 (0.26–0.82)	0.008
University or higher	0.43 (0.20–0.90)	0.025	0.46 (0.13–1.64)	0.230	0.34 (0.13–0.89)	0.028	0.47 (0.16–1.35)	0.160	0.42 (0.14–1.24)	0.117
Financial strain										
No	1		1		1		1		1	
Yes	1.09 (0.75–1.56)	0.661	0.84 (0.50–1.41)	0.518	1.50 (0.89–2.54)	0.131	0.98 (0.58–1.66)	0.945	1.11 (0.66–1.89)	0.690
Living area										
Rural	1		1		1		1		1	
Urban	0.75 (0.51–1.09)	0.135	0.75 (0.43–1.30)	0.306	0.83 (0.48–1.54)	0.518	0.88 (0.52–1.50)	0.644	0.63 (0.35–1.11)	0.110
Living with spouse										
No	1		1		1		1		1	
Yes	0.90 (0.60–1.35)	0.615	0.89 (0.49–1.59)	0.689	0.97 (0.54–1.74)	0.909	0.52 (0.28–0.94)	0.031	1.46 (0.82–2.60)	0.195
Living with children										
No	1		1		1		1		1	
Yes	1.06 (0.73–1.52)	0.771	0.93 (0.55–1.56)	0.772	1.26 (0.75–2.13)	0.385	0.99 (0.59–1.66)	0.961	1.06 (0.63–1.81)	0.822

^1^ Interaction using communication devices was defined as the use of tools of telephone, e-mail, or social media to communicate with family and others; ^2^ Interaction not using communication devices was defined as face-to-face direct interactions with family and others at social gatherings, such as going out together or visiting each other’s homes; IADL, Instrumental Activities of Daily Living.

## Data Availability

The data presented in this study are available on request from the corresponding author. The data are not publicly available due to them containing information that could compromise the privacy/consent of research participants.
